# Signaling via the p75 neurotrophin receptor facilitates amyloid-β-induced dendritic spine pathology

**DOI:** 10.1038/s41598-020-70153-4

**Published:** 2020-08-07

**Authors:** Abhisarika Patnaik, Marta Zagrebelsky, Martin Korte, Andreas Holz

**Affiliations:** 1 Zoological Institute, Division of Cellular Neurobiology, TU Braunschweig, 38108 Braunschweig, Germany; 2 Group Neuroinflammation and Neurodegeneration, Helmholtz Centre for Infection Research, AG NIND, Inhoffenstrasse 7, 38124 Braunschweig, Germany

**Keywords:** Cellular neuroscience, Diseases of the nervous system, Neurodegeneration, Alzheimer's disease, Neuroscience

## Abstract

Synapse and dendritic spine loss induced by amyloid-β oligomers is one of the main hallmarks of the early phases of Alzheimer’s disease (AD) and is directly correlated with the cognitive decline typical of this pathology. The p75 neurotrophin receptor (p75^NTR^) binds amyloid-β oligomers in the nM range. While it was shown that µM concentrations of amyloid-β mediate cell death, the role and intracellular signaling of p75^NTR^ for dendritic spine pathology induced by sublethal concentrations of amyloid-β has not been analyzed. We describe here p75^NTR^ as a crucial binding partner in mediating effects of soluble amyloid-β oligomers on dendritic spine density and structure in non-apoptotic hippocampal neurons. Removing or over-expressing p75^NTR^ in neurons rescues or exacerbates the typical loss of dendritic spines and their structural alterations observed upon treatment with nM concentrations of amyloid-β oligomers. Moreover, we show that binding of amyloid-β oligomers to p75^NTR^ activates the RhoA/ROCK signaling cascade resulting in the fast stabilization of the actin spinoskeleton. Our results describe a role for p75^NTR^ and downstream signaling events triggered by binding of amyloid-β oligomers and causing dendritic spine pathology. These observations further our understanding of the molecular mechanisms underlying one of the main early neuropathological hallmarks of AD.

## Introduction

The p75 neurotrophin receptor (p75^NTR^; also known as Nerve Growth Factor Receptor, NGFR) is a member of the tumor necrosis factor receptor superfamily that is capable to bind all neurotrophins with similar affinities in the nano-molar scale^1^. Originally, this transmembrane protein was described to act in concert with the frequently co-expressed Trk receptor family of tyrosine kinases to balance neuronal survival and death, in particular during development^2^. However, p75^NTR^ itself has also been found to serve multiple, in parts opposing cellular functions within the CNS and in the periphery^1^. For instance, triggered by neurotrophins and their proforms, the p75^NTR^ signaling molecule can induce apoptosis in neural cells^3^, but is also able to promote cell survival in different cellular settings^4,5^. Moreover, p75^NTR^ has been implicated in cell cycle control^6,7^, axonal outgrowth^8^, synaptic transmission as well as functional and structural plasticity^9–11^.

Interestingly, p75^NTR^ serves as a receptor for amyloid-β, a major component of the plaques found in the brain of Alzheimer’s disease (AD) patients^12^. The extracellular domain of p75^NTR^ is able to bind Aβ-peptides at effective concentrations similar to those of NGF^13^. The interaction between p75^NTR^ and amyloid-β in the µM range has been shown to cause cytotoxicity and apoptosis in different cell types^12,14^, including hippocampal neurons^15,16^. In addition, also Tau pathology might be influenced by p75^NTR^^17,18^. These studies are in agreement with early findings showing that either the overexpression or the induced upregulation of the p75^NTR^ receptor enhanced amyloid-β-triggered cytotoxic effects on neuronal cells^19^. However, p75^NTR^ also has been reported to confer neuroprotective capabilities against the damaging actions of soluble Aβ oligomers^20,21^ as p75^NTR^/Aβ_1–42_ interactions triggered rapid neurite formation^22^.

Additional findings link p75^NTR^ with neurodegeneration and AD. For instance, while the expression of p75^NTR^ diminishes in the CNS once development is completed, adult cholinergic neurons of the basal forebrain, which are among the first CNS neurons affected in AD^23^, persistently express high levels of p75^NTR^^24^. Moreover, p75^NTR^ expression is upregulated in neurons of the aged and AD-afflicted cortex^25^.

While most reported studies analyzed neural apoptosis mediated by the binding of amyloid-β to p75^NTR^, we sought to examine the acute effects triggered by p75^NTR^/amyloid-β interactions with a specific focus on synapses and dendritic spine pathology. Indeed, amyloid-β oligomers are binding preferentially to synapses at dendritic spines^26^ and rapidly induce dendritic spine loss^27^. The loss of dendritic spines observed in AD is intimately linked with synaptic dysfunction and loss of memory and cognition^28^. Furthermore, p75^NTR^ has been shown to negatively modulate dendrite complexity and dendritic spine density in hippocampal neurons^9^.

We took advantage of a series of loss- and gain-of-function approaches for p75^NTR^ including a knockout (ko) mouse strain having a complete p75^NTR^ deletion^29^ and its over-expression. Comparing mature primary hippocampal neurons from wild type controls with those from p75^NTR^ ko mice we observe an almost complete protection from Aβ_1–42_-induced changes in dendritic spine number and morphology in knockout animals. On the contrary, gain-of-function for p75^NTR^ exacerbates the spine alterations seen in wild type neurons. Analyzing the downstream-signaling events triggered by the amyloid-β/p75^NTR^ interaction, we implicate RhoA/ROCK activation as an important factor in causing actin cytoskeleton stabilization presumably mediating the morphological alterations at dendritic spines.

## Results

### The genetic ablation of the p75 neurotrophin receptor protects hippocampal neurons against Aβ_1–42_ mediated spine pathology

It is well established that the long-term treatment of both cell lines and primary neurons with amyloid-β (Aβ_1–42_) oligomers in the µM range results in neuronal degeneration and cell death per apoptosis^30^. These effects have been shown to depend on the specific binding of the Aβ_1–42_ oligomers to the p75^NTR^ receptor^14,16^ and the activation of downstream signaling. Here we were primarily interested in analyzing the sublethal effects of acute Aβ_1–42_/p75^NTR^ interactions in the nM range. By this means we analyzed possible intracellular signaling events which might lead to dendritic spine alterations. Dendritic spine pathology is one of the principal symptoms of Aβ_1–42_ accumulation in early AD.

**Figure 1:**
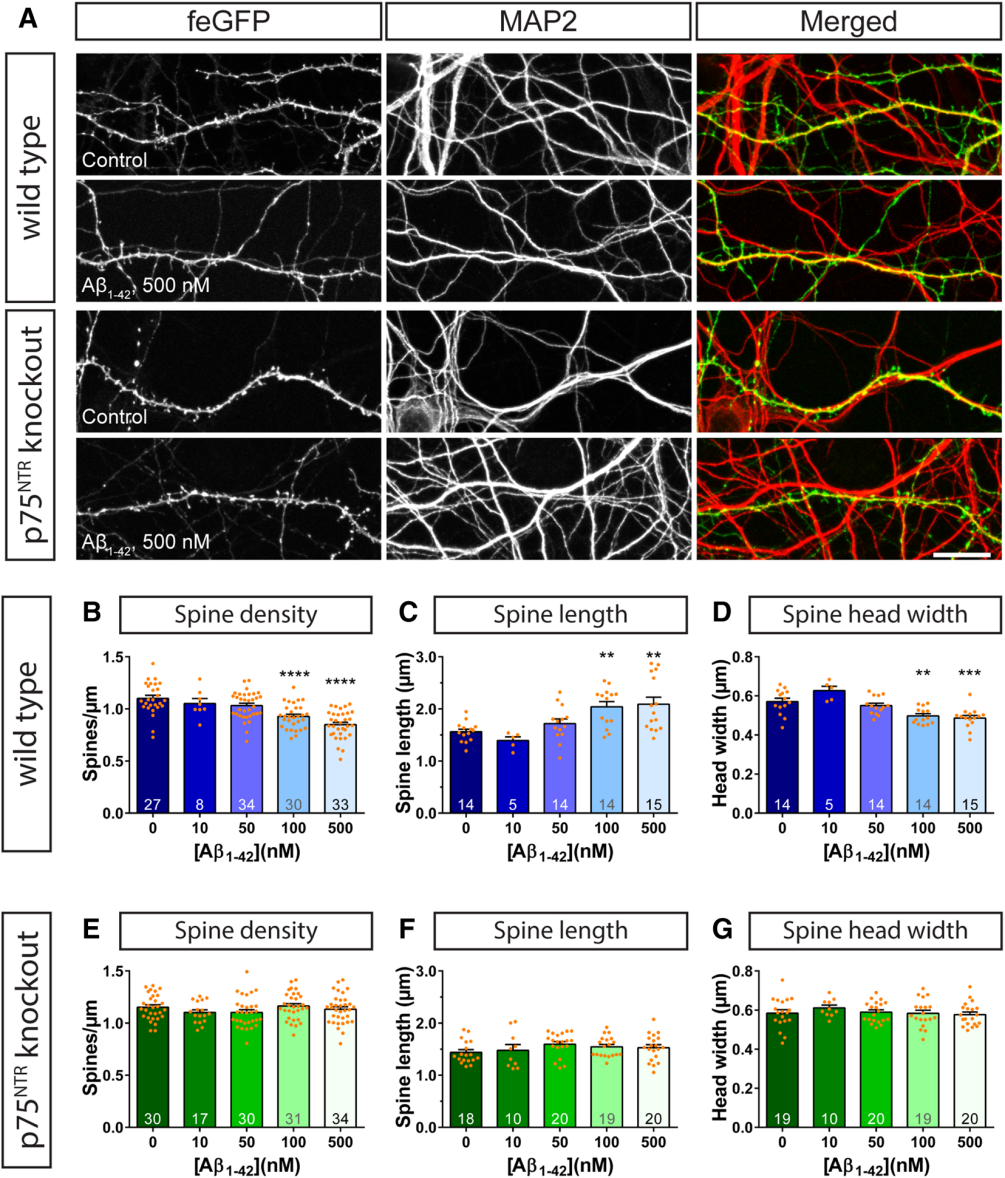
The ablation of p75^NTR^ rescues acute amyloid-β induced changes in dendritic spine morphology. Primary hippocampal neurons from wild type (**A**, **B**–**D**) and p75^NTR^ ko (**A, E–G**) mice were incubated with vehicle (PBS; 0) or with 10, 50, 100, or 500 nM Aβ_1–42_ oligomers for 6 h. (**A**) shows representative images as maximum intensity projections of dendrites of f-GFP^+^ expressing neurons, which were co-stained with MAP2 to visualize the entire dendritic network. An evaluation of the spine density (**B**, **E**), spine length (**C**, **F**), and spine head width (**D**, **G**) is shown in the graphs where dendritic segments with a length of ≥ 100 µm were used for analysis for wild type and p75^NTR^ ko neurons. Columns represent mean values + SEM and dots mark data points of individual neurons. The number of evaluated cells, obtained from three independent sets of experiments, is given in each column of the graphs. Statistical significance was tested using a one-way ANOVA with Sidak post-test. ** = p < 0.01, *** = p < 0.001, **** = p < 0.0001. Scale bar: 20 µm.

Initially, f-eGFP expressing DIV14 primary hippocampal neurons, obtained from C57BL/6 wild type animals were incubated with increasing amounts of Aβ_1–42_ in the nM range and their impact on dendritic spine density and morphology was analyzed after six hours (Fig. 1A–D). This time point has been chosen since it has been reported previously to cause significant dendritic alterations upon treatment with Aβ oligomers^31^. While incubations with 10 and 50 nM Aβ_1–42_ oligomers resulted in no or only a mild reduction in dendritic spine density when compared to the control condition, application of 100 nM Aβ_1–42_ oligomers resulted in a significantly lower dendritic spine density (Fig. 1B). This decrease was even stronger upon application of 500 nM Aβ_1–42_ oligomers (Fig. 1B), indicating a dose-dependent effect. The decrease in spine density was associated with structural changes in the remaining dendritic spines. Particularly, a significant increase in dendritic spine length (Fig. 1C) and a significant decrease in the spine head width (Fig. 1D) was observed using the higher concentrations of Aβ_1–42_ oligomers (100–500 nM). Interestingly, while the application of 50 nM Aβ_1–42_ oligomers did not alter dendritic spine structure, 10 nM Aβ_1–42_ oligomers caused a small increase in the spine head width (Fig. 1D) that coincided with a decrease in the length (Fig. 1C) of treated spines.

**Figure 2:**
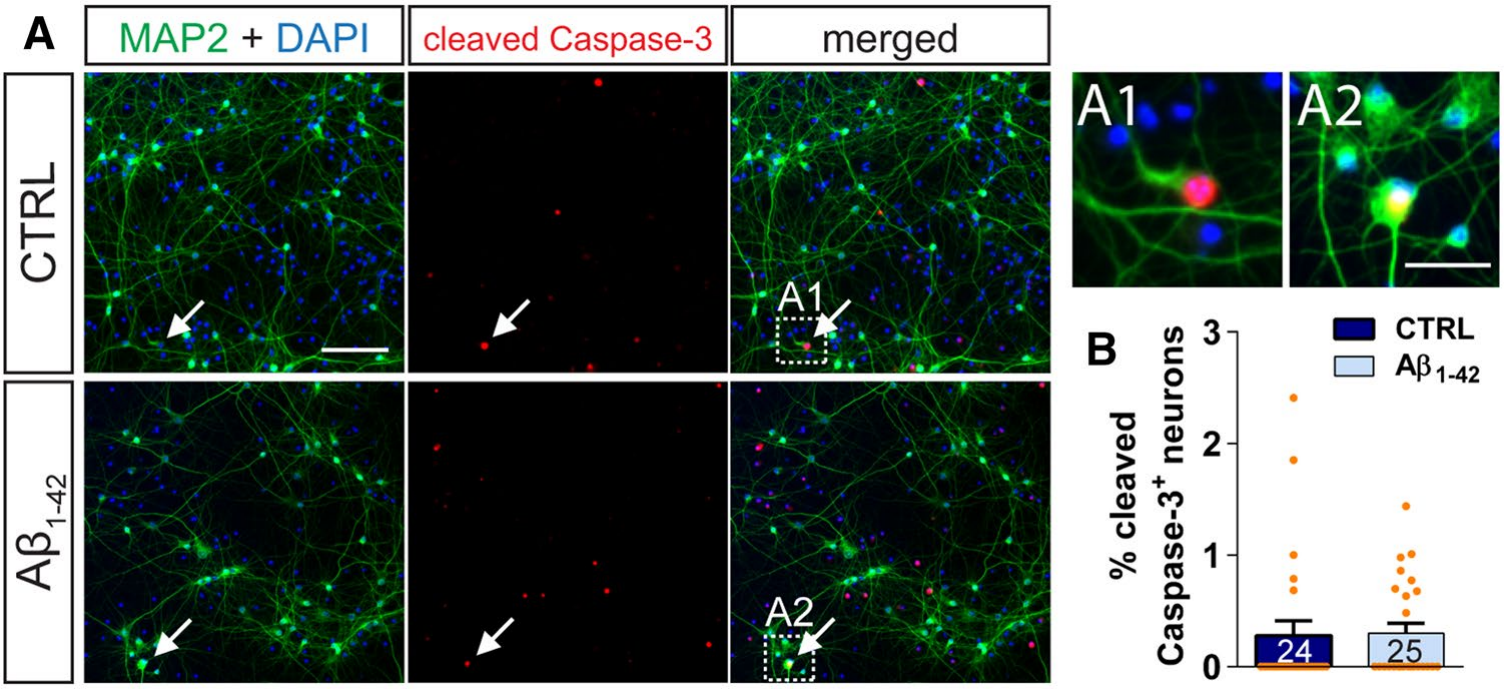
Treatment with nM amyloid-β does not induce apoptosis in hippocampal neurons. (**A**) Shows representative images of primary hippocampal cultures from wild type that were treated with vehicle (CTRL, top panels) or with 500 nM Aβ_1–42_ oligomers (bottom panels) for 6 h and were stained for MAP2 (green) and cleaved caspase-3 (red). A DAPI staining visualizes nuclei (blue). Arrows indicate MAP2-expressing neurons positive for the cleaved caspase-3 (Scale bar: 100 µm). The inserts A1 and A2 show in detail two neuronal cell bodies positive for the cleaved caspase-3 (Scale bar: 20 µm). (**B**) The graph compares the percent of neurons positive for the cleaved caspase-3 in vehicle-treated (dark blue) and Aβ_1–42_ oligomers treated (light blue) wild type hippocampal cultures. Columns represent mean values + SEM. The number of evaluated fields, obtained from two coverslips of two independent sets of experiments, is given within each column of the graphs. Statistical significance was tested using a Student’s t-test (p = 0.8903).

Importantly, a 6-h treatment with 500 nM Aβ_1–42_ oligomers did not cause any detectable apoptosis in neurons (Fig. 2, Supplementary Fig. S1). In general, only very few activated Caspase-3 (Fig. 2) and TUNEL-positive (Fig. S1) neurons (defined by their MAP2 expression; Figs. 2A and S1A) were observed in the analyzed primary hippocampal cultures and there were no differences in apoptotic neurons between vehicle- and Aβ_1–42_-treated neuronal cultures (Figs. 2B and S1B–D).

In summary, these data demonstrate that a 6-h treatment of wild type mature primary hippocampal neurons with Aβ_1–42_ oligomers causes a reduction in dendritic spine density associated with a switch toward an immature phenotype of the remaining dendritic spines. These observations are in line with previous reports^27^. Interestingly, low concentrations of Aβ_1–42_ (10 nM) seem to promote dendritic spine maturation. It should be noted that a rather brief incubation for 15 min of primary hippocampal neurons with amyloid-β oligomers had no effect on both the density and the morphology of dendritic spines (Supplementary Fig. S2).

Intriguingly, when increasing amounts of Aβ_1–42_ oligomers were added to f-eGFP expressing mature primary hippocampal neurons obtained from p75^NTRexon-IV^ knockout mice^29^, the alterations observed in wild type mice were completely prevented (Fig. 1A, E–G, Supplementary Fig. S2). In fact, when compared to the control conditions even at the highest concentration of Aβ_1–42_ oligomers tested (i.e. 500 nM) no changes in dendritic spine density (Fig. 1E) as well as in dendritic spine length (Fig. 1F) and head width (Fig. 1G) were detected. Considering that p75^NTR^ is not the only reported neuronal receptor serving as a ligand for amyloid-β^32^, these results indicate that it must play a major role in triggering Aβ_1–42_-induced alterations in dendritic spine morphology.

### Overexpression of p75^NTR^ leads to higher sensitivity of primary hippocampal neurons to the deleterious effects of Aβ_1–42_ on dendritic spines

**Figure 3:**
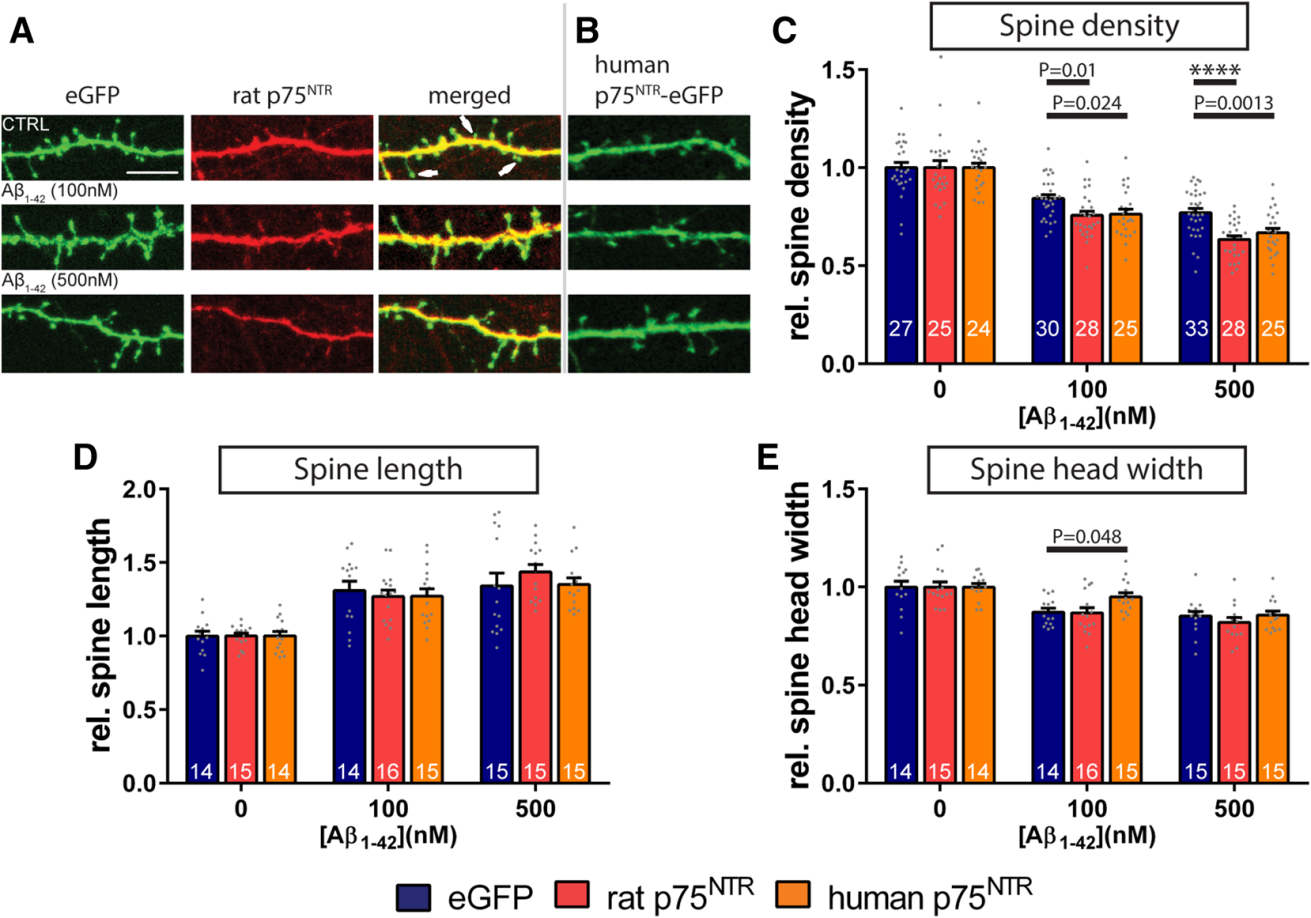
Overexpression of the p75^NTR^ signaling receptor aggravates the dendritic spine loss triggered by amyloid-β_1–42_ oligomers. Primary hippocampal cultures were either transfected with expression vectors encoding f-eGFP (eGFP, **C**–**E**) or with a fusion protein of human full-length p75^NTR^ with eGFP (human p75^NTR^-eGFP, **B**, **C**–**E**), or alternatively were co-transfected with plasmids encoding f-eGFP and full-length rat p75^NTR^ (rat p75^NTR^, **A**, **C**–**E**). (**A**) demonstrates the co-expression of f-eGFP (green, left panel) with immunohistochemically-labeled rat p75^NTR^ (red, middle panel) in a dendritic segment of a transfected neuron. Note that p75^NTR^ overexpression is not noticeable in all dendritic spines (arrows in the right, ‘merged’ panel). (**B**) shows the overexpression of the human p75^NTR^ variant that is directly fused to eGFP. Subsequent to all transfections, the neurons (DIV14) either received vehicle (CTRL) or were incubated with increasing concentrations of Aβ_1–42_ oligomers as indicated for 6 h (**C**–**E**). Amyloid-β treated samples of each experimental group were normalized to the corresponding controls, which were treated with equivalent volumes of vehicle as control. The evaluation of dendritic spine density (**C**), spine length (**D**), and spine head width (**E**) is shown in the graphs. The data are presented as mean + SEM. One-way ANOVA with Sidak post-test was used to compare the means of the control (eGFP) to p75^NTR^ overexpression groups for each Aβ_1–42_-treatment. P values as indicated or **** = p < 0.0001. The number of analyzed neurons resulting from three independent experiments is given for each data column. Scale bar: 5 µm.

Next, we evaluated the impact of the neuronal overexpression of full-length variants of p75^NTR^ on mediating amyloid-β induced dendritic changes. For this purpose, primary hippocampal neurons were either transfected with an expression vector encoding the rat p75^NTR^ together with f-eGFP to visualize the neurons (Fig. 3A)^9^ or with the eGFP-tagged human p75^NTR^ (Fig. 3B)^33^. As a control, neuronal cultures were transfected with the f-eGFP-expressing plasmid alone. Subsequently, the neuronal cultures were treated on DIV14 with increasing concentrations of Aβ_1–42_ oligomers for 6 h and the spine density and morphology of individual neurons were evaluated. Since the sole over-expression of p75^NTR^ causes alterations in dendritic spine morphology^9^, the results obtained from amyloid-β treated dendrites of each experimental group were normalized to the corresponding control samples without amyloid-β stimulation (Fig. 3C–E).

Similar to the results described above in Fig. 1, the neurons that expressed f-eGFP alone and were treated with Aβ_1–42_ oligomers showed a dose-dependent decrease in dendritic spine density when compared to vehicle controls (Fig. 3C). Likewise, we observed an increase in spine length (Fig. 3D) as well as a decrease in spine head width (Fig. 3E) in those neurons that had received amyloid-β oligomers. Remarkably, the overexpression of both human and rodent p75^NTR^ isoforms led to a significantly stronger decrease in the density of dendritic spines after both 100 and 500 nM amyloid-β treatment when compared to f-eGFP-expressing control neurons, which had been treated with identical amounts of amyloid-β oligomers (Fig. 3C). The application of Aβ_1–42_ oligomers, however, had no comparable effects on the length (Fig. 3D) nor on the head width (Fig. 3E) of the remaining dendritic spines of p75^NTR^ overexpressing neurons.

### The localization of amyloid-β at dendritic spines is dependent on the levels of p75^NTR^

**Figure 4:**
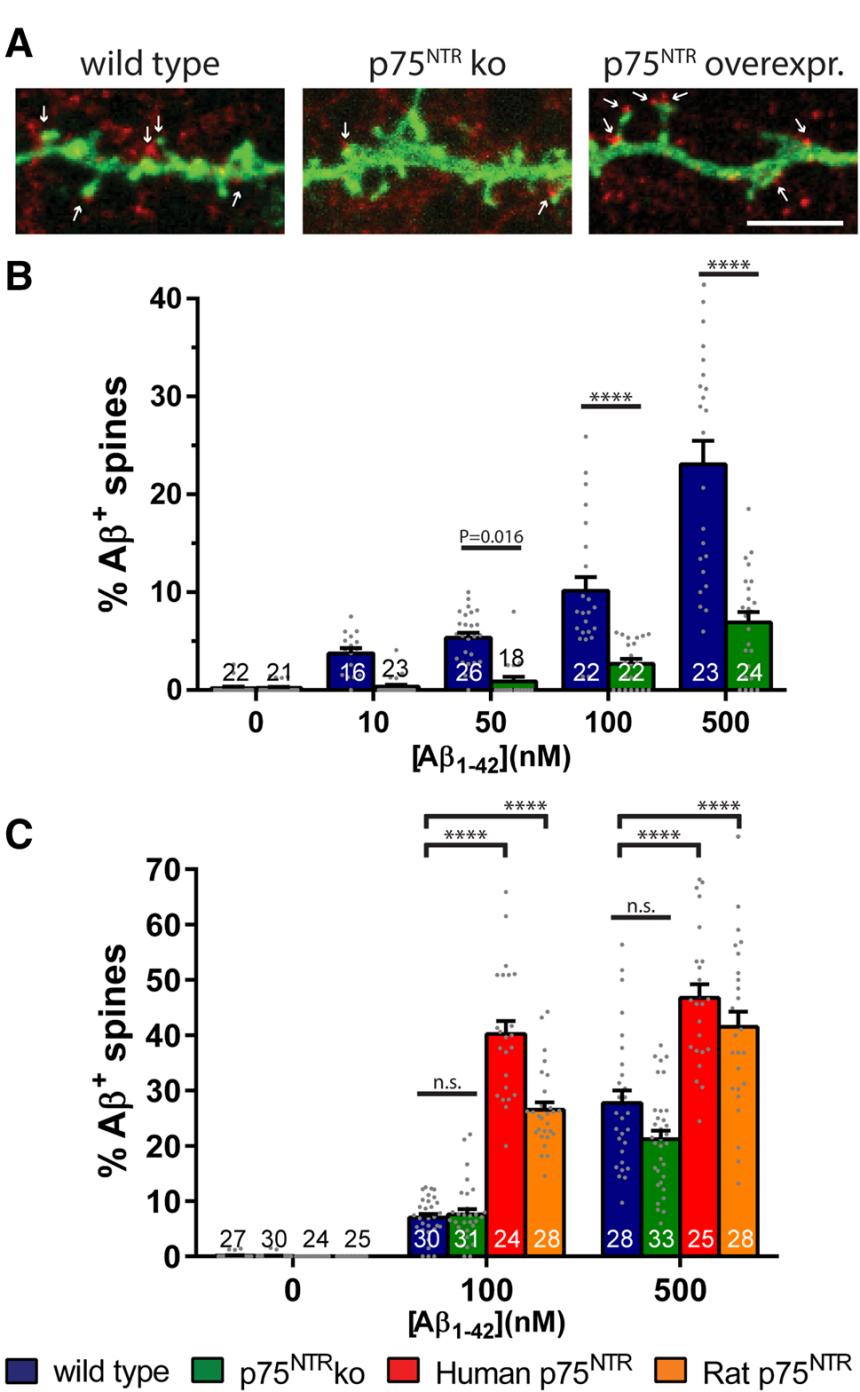
Colocalization of amyloid-β to dendritic spines is dependent on the expression levels of p75^NTR^. (**A**) gives a representative example of the distribution of amyloid-β immunoreactive puncta (red) at a dendritic segment of f-eGFP-transfected neurons (green), which either were obtained from wild type (left) or p75^NTR^ ko (middle) mice, or were co-transfected with an expression vector encoding rodent p75^NTR^ (right). Arrows indicate dendritic spines colocalized with amyloid-β-immunoreactive puncta. The red puncta that did not colocalize with dendritic f-eGFP labeled spines stem from out-of-focus amyloid-β oligomers binding to neuronal processes of non-transfected neurons. Treatment of the primary hippocampal cultures (DIV 14) with Aβ_1–42_ oligomers was for 15 min. The graphs illustrate the quantification of the proportion of amyloid-β-positive dendritic spines of neurons, which received vehicle or increasing amounts of Aβ_1–42_ oligomers for 15 min (**B**) or 6 h (**C**). Hippocampal neurons were wild type (**B**, **C**; blue), had knockout for p75^NTR^ (**B**, **C**; green), or overexpressed human (**C**, red) or rat p75^NTR^ (**C**, orange). Each bar shows the mean values + SEM. To compare the means from wild type and p75^NTR^ knockout neurons in B, One-way ANOVA with Sidak post-test was used. In C, a Two-way ANOVA with Tukey post-test was applied. The numbers at the bottom of each column give the amount of analyzed neurons, which were obtained from three independent experiments. Statistical significances as indicated or **** = p < 0.0001; ns = not significant. Scale bar in (**A**) is equivalent to 5 µm.

Our data suggest that p75^NTR^ is an important membrane component mediating the effects of Aβ_1–42_ oligomers on dendritic spines. Hence, we sought to evaluate in more detail the direct interactions of amyloid-β with dendritic spines depending on the p75^NTR^ expression levels. Initially, we compared the colocalization of amyloid-β-immunoreactive puncta with dendritic spine heads in f-eGFP expressing wild type and p75^NTR^ knockout primary hippocampal neurons after a short-term incubation of 15 min with increasing concentrations of Aβ_1–42_ oligomers (Fig. 4A,B). We observed that both in wild type and p75^NTR^ ko neurons the number of amyloid-β-immunoreactive puncta associated with dendritic spines increased in a concentration-dependent manner. In addition, the percent of spines colocalized with amyloid-β-immunoreactive puncta in wild type neurons was consistently higher when compared to p75^NTR^ ko neurons (Fig. 4B). At the highest concentration of Aβ_1–42_ oligomers analyzed (i.e. 500 nM Aβ_1–42_), 23.1 ± 2.4% (mean ± SEM; n = 23) of wild type dendritic spines were associated with amyloid-β-immunoreactive puncta, whereas in contrast spines from p75^NTR^ ko neurons showed a colocalization ratio of only 6.9 ± 1.1% (mean ± SEM; n = 24), a difference that was statistically highly significant (p < 0.0001).

In order to analyze more long-term effects of the interaction of Aβ_1–42_ oligomers with dendritic spines, we extended the Aβ_1–42_ incubation times to 6 h (Fig. 4C). At this time point, the differences in the relative amounts of spine-associated amyloid-β-immunoreactive puncta between wild type and p75^NTR^ ko neurons were less pronounced and did not reach any longer statistical significance (27.7 ± 2.3%, n = 28 vs. 21.2 ± 1.5%, n = 33, respectively, for treatment with 500 nM Aβ_1–42_; mean ± SEM, p = 0.08, Fig. 4C). The overexpression of rodent or human p75^NTR^ in neurons, however, significantly augmented the amount of amyloid-β-immunoreactive puncta localized at dendritic spines (Fig. 4C) when compared to wild type. Interestingly, the overexpression of the human p75^NTR^ resulted in a higher colocalization than the rodent one (Fig. 4C). This data emphasizes that p75^NTR^ serves as an important receptor for amyloid-β binding, in particular for the initial phases of amyloid-β/receptor interactions. However, it also confirms the notion that other amyloid-β receptors are located at dendritic spines of hippocampal neurons.

### Mechanistic insight: amyloid-β induced activation of RhoA requires presence of p75^NTR^

We documented amyloid-β-induced alterations of dendritic spines of primary neurons that could be rescued by the removal of p75^NTR^. Moreover, we show that Aβ_1–42_ is localized at dendritic spines and that the levels of neuronal p75^NTR^ expression influence strongly the association of amyloid-β with spines. Therefore, we next searched for a potential downstream mechanism explaining the amyloid-β/p75^NTR^ mediated changes in spine morphology. Since it has been reported that p75^NTR^ is capable to activate RhoA^8^ at a fast time scale^34^ and RhoA signaling is implicated in remodeling the actin cytoskeleton, including the modulation of spine dynamics^35,36^ within minutes^37^, we hypothesized that Aβ_1–42_/p75^NTR^ interactions trigger the activation of the RhoA/ROCK pathway, ultimately leading to changes in the actin composition and dendritic spine architecture.

**Figure 5:**
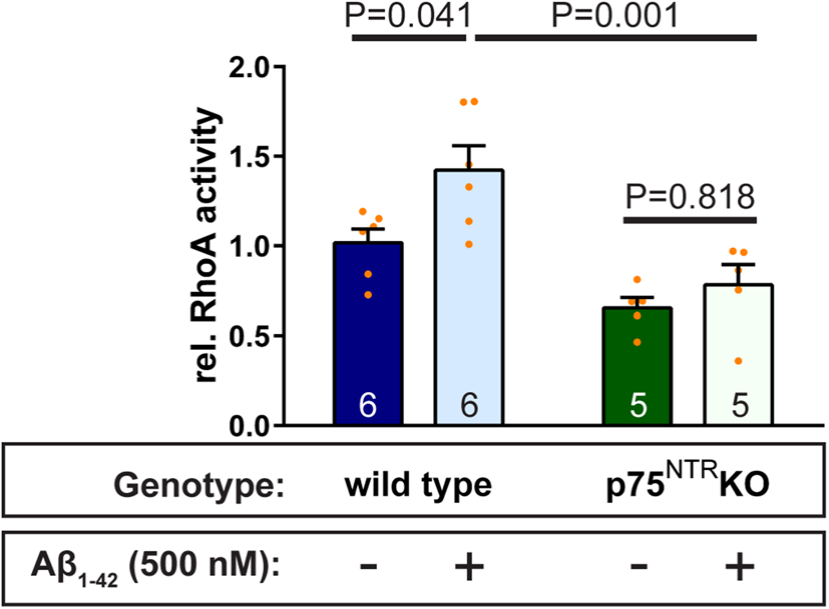
Interactions of Aβ_1–42_ oligomers with the p75^NTR^ transmembrane receptor causes activation of cytoplasmic RhoA. The treatment of primary hippocampal neurons (DIV14) with vehicle vs. 500 nM Aβ_1–42_ oligomers for 10 min results in a significant activation of the small GTPase RhoA in p75^NTR^ competent cells. In contrast, neurons obtained from p75^NTR^ ko mice did not show a comparable activation of RhoA in response to an equivalent amyloid-β treatment. RhoA activity was determined in neuronal preparations by G-ELISA. Columns values are mean + SEM. Statistical analysis was performed using a Two-way ANOVA assay with Tukey post-test. P values are specified. The number of experimental repetitions is given in the graphs.

We first analyzed if amyloid-β is capable to activate RhoA in murine primary hippocampal cultures and whether this cellular event is influenced by the presence of p75^NTR^. Hence, we added Aβ_1–42_ oligomers for 10 min to primary neurons from wild type and from p75^NTR^ ko cultures and performed an ELISA-based assay to determine the activation state of RhoA molecules, i.e. its GTP-bound form^38^. Intriguingly, we observed a significant activation of RhoA for wild type hippocampal cultures upon treatment with Aβ_1–42_ oligomers (Fig. 5). Interestingly, the RhoA activation level in vehicle-treated p75^NTR^ ko cultures was lower, albeit not significantly (p = 0.0961, Two-way ANOVA with Tukey post-test), than in vehicle-treated wild type neurons (Fig. 5). Importantly, the application of Aβ_1–42_ oligomers to p75^NTR^-deficient neuronal cultures did not result in any measurable RhoA activation (Fig. 5). This data indicate that Aβ_1–42_ oligomers are able to cause the p75^NTR^-dependent activation of RhoA in primary hippocampal cultures.

### The inhibition of p75^NTR^-mediated RhoA/ROCK signaling rescues amyloid-induced changes in dendritic spine morphology

**Figure 6:**
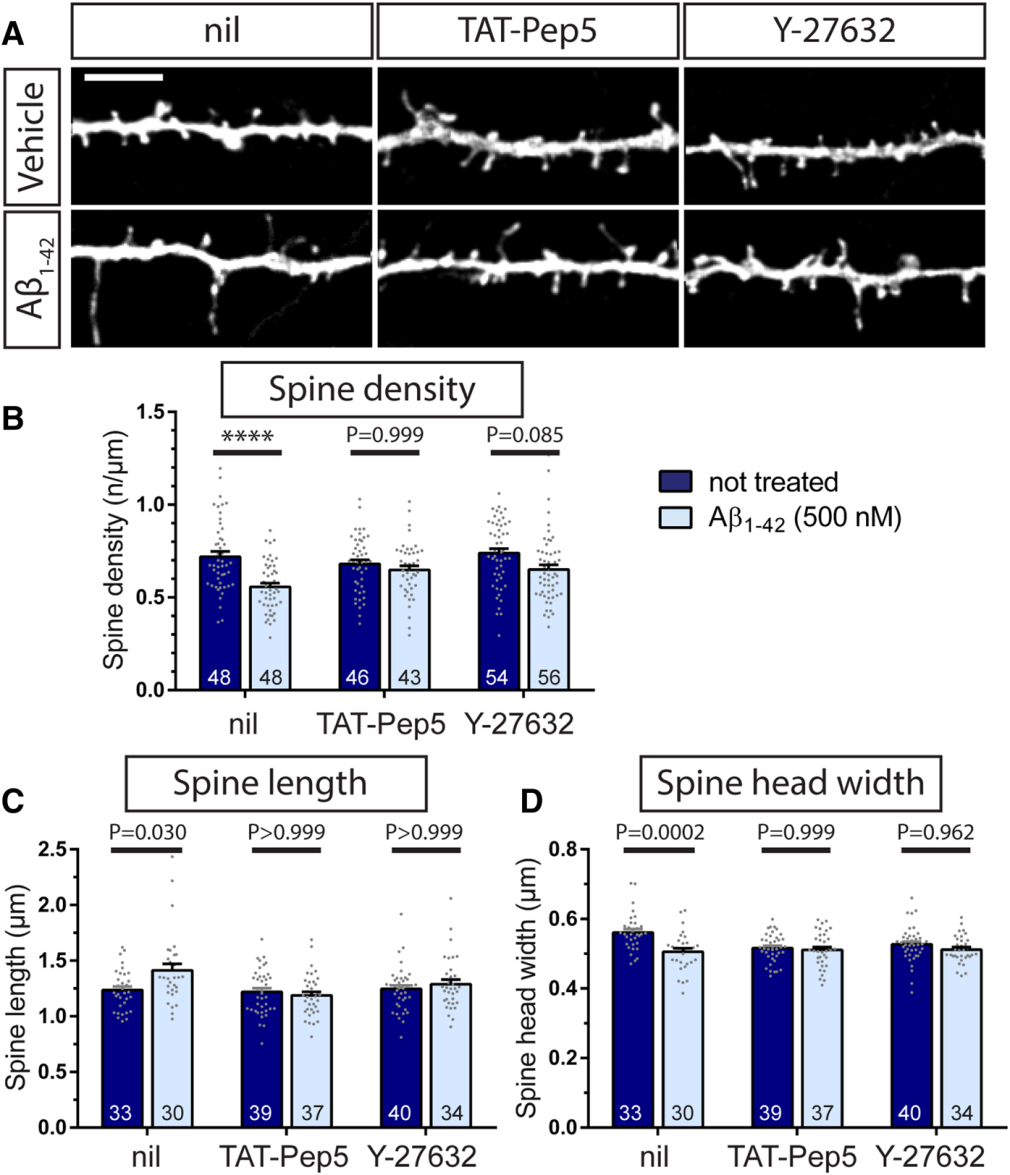
The inhibition of p75^NTR^-mediated RhoA/ROCK signaling protects from Aβ-induced morphological changes. The TAT-Pep5 peptide was added to primary hippocampal cultures (DIV 14) to inhibit p75^NTR^–mediated intracellular RhoA activation and the Y-27632 inhibitor was applied to prevent ROCK activation. Some neuronal cultures were incubated with vehicle as control (nil). Subsequently, neuronal cultures were incubated with vehicle (Vehicle) or with 500 nM Aβ_1–42_ oligomers (Aβ_1–42_) for 6 h. (**A**) depicts representative dendritic segments of f-eGFP-transfected neurons treated as indicated (scale bar = 5 µm). The quantification of the effect of amyloid-β treatment on spine density (**B**), spine length (**C**), and spine head width (**D**) is shown in the graphs. Each column gives the mean value + SEM where the numbers indicate the analyzed neurons (N = 3). For statistical evaluation, a One-way ANOVA with Sidak post-test was used to compare the means of each of the control to the corresponding amyloid-β groups. P values as indicated or **** = p < 0.0001.

Next, we utilized inhibitors known to prevent the activation of p75^NTR^ downstream signaling to RhoA to test the hypothesis that p75^NTR^ is able to mediate the effect of Aβ_1–42_ oligomers on dendritic spine structure by directly controlling the activation of the RhoA. The TAT-Pep5 fusion peptide has been described to be able to enter the cell and to specifically inhibit p75^NTR^-mediated RhoA activation^39^. In addition, we also employed the Rho-associated coiled-coil containing protein kinase (ROCK) inhibitor Y-27632^40^. Upon a 6-h treatment with 500 nM Aβ_1–42_ oligomers, f-eGFP expressing primary hippocampal neurons showed a significant decrease in dendritic spine density (Fig. 6A,B) associated with significant morphological changes in spine length (Fig. 6A,C) and spine head width (Fig. 6A,D), similar to the results described above (see Fig. 1). Interestingly, a pretreatment of the neuronal cultures either with the TAT-Pep5 peptide, inhibiting the p75^NTR^-mediated RhoA activation, or with the pan-ROCK inhibitor Y-27632 both protected hippocampal neurons from the Aβ_1–42_ oligomer-induced alterations in dendritic spine number and structure (Fig. 6A–D). Specifically, preventing the p75^NTR^ mediated activation of RhoA or inhibiting ROCK blocked significant changes in either dendritic spine density (Fig. 6A,B), spine elongation (Fig. 6A,C), or spine head diameter (Fig. 6A,D) when compared to the corresponding controls.

### Involvement of p75^NTR^ amyloid-β induced changes in the actin filament

Above we analyzed the contribution of the RhoA/ROCK pathway in transducing the interaction of Aβ_1–42_ oligomers with the p75^NTR^ receptor. Finally, we examined whether these p75^NTR^ mediated signaling events influenced the actin cytoskeletal network in dendritic spines.

**Figure 7:**
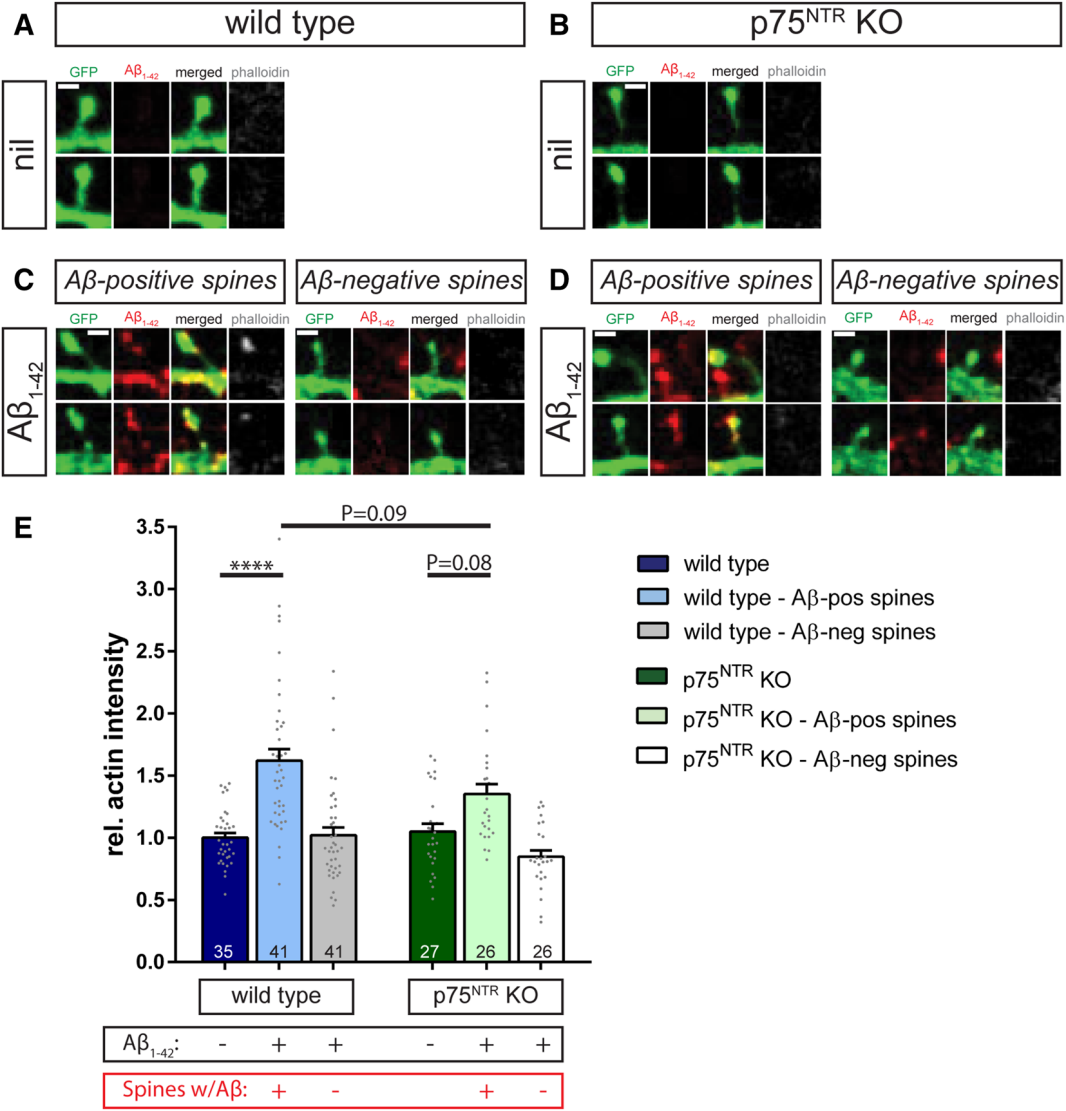
Aβ_1–42_/p75^NTR^ interactions influence actin polymerization. Individual dendritic spines are shown from f-eGFP transfected (green) wild type (**A**, **C**) and p75^NTR^ ko (**B**, **D**) neurons treated for 10 min with 500 nM Aβ_1–42_ oligomers (**C**, **D**) or vehicle (**A**, **B**). Presence of amyloid-β (red) and F-actin (phalloidin staining in grey) was visualized by fluorescent confocal microscopy. (**C**) and (**D**) show representative examples of dendritic spines with and without amyloid-β colocalization, i.e. Aβ-positive and Aβ-negative spines, respectively. A quantification of these results is plotted in (**E**), where each column represents the mean values and the error bars the SEM (numbers indicate the neurons used for evaluation, N = 3). A Two-way ANOVA with Tukey post-test was applied to determine statistical significance. **** = p < 0.0001, relevant P values are as indicated in the graph. Scale bars in (**A**) and (**B**) = 1 µm.

First, we used phalloidin staining to compare the levels of F-actin within dendritic spines from individual wild type and p75^NTR^ knockout animals (Fig. 7A,B). Under basal conditions, only low levels of F-actin could be identified in the dendritic spines of both genotypes (Fig. 7A,B).

Next, we sought to assess the effect of the application of Aβ_1–42_ oligomers on the F-actin levels within spines by incubating f-eGFP expressing primary hippocampal neurons with 500 nM Aβ_1–42_ for 10 min. We observed that this brief treatment caused a significant increase in the F-actin intensity specifically in those dendritic spines colocalized with Aβ_1–42_ immunoreactive puncta (Fig. 7C,E). Importantly, the levels of F-actin within dendritic spines were strictly dependent on the presence of Aβ_1–42_ since dendritic spines of the same neurons lacking Aβ_1–42_ immunoreactive puncta did not show any significant increase in F-actin (Fig. 7C,E). Interestingly, in p75^NTR^-deficient neurons the F-actin intensity within amyloid-β^+^ dendritic spines increased only slightly, but not significantly, when compared to the control condition (p = 0.08, Fig. 7D,E). Furthermore, the F-actin intensity in spines of p75^NTR^ ko neurons was clearly, albeit also not significantly, lower than in wild type cells (p = 0.09; Fig. 7E).

In summary, our data suggest that the interaction of Aβ_1–42_ oligomers with the p75^NTR^ triggers an intracellular RhoA/ROCK signaling cascade that likely leads to alterations in actin polymerization in dendritic spines of primary hippocampal neurons. The ablation of p75^NTR^ largely prevents the activation of these p75^NTR^ mediated intracellular signaling events of the RhoA/ROCK pathway and the actin polymerization, thereby protecting p75^NTR^ ko neurons from the Aβ_1–42_-induced dendritic spine pathology.

## Discussion

Understanding the role of oligomeric Aβ_1–42_ in inducing the dendritic spine pathology most likely causing the symptoms typical of the initial phases of AD requires to elucidate the signaling mechanisms activated by amyloid-β at synapses. While Aβ has been shown to bind to several membrane receptors, our results provide strong evidence for a crucial role of p75^NTR^ signaling in the Aβ_1–42_-induced structural alterations at synapses directly. In this study, we show that p75^NTR^ signaling is required for the dendritic spine loss and structural changes observed in mature primary hippocampal neurons upon application of increasing nM concentrations of Aβ_1–42_ oligomers. Moreover, we show that the activation of p75^NTR^ upon Aβ_1–42_ oligomer treatment results in a RhoA-ROCK-mediated stabilization of the actin spinoskeleton followed by spine loss as well as shrinking and elongation of the remaining spines.

While Aβ plaques are the typical hallmark of the advanced phases of AD it is now rather believed that it is the soluble Aβ_1–42_ oligomers that, binding with high affinity at synaptic sites, impair the function and structure of synapses at the onset of the disease^41^. Soluble Aβ_1–42_ oligomers are found in several AD mouse models and in human patients and their levels correlate with synaptic loss^42^, impairment of synaptic transmission and plasticity^43,44^ as well as AD-associated cognitive decline^45,46^. While in vitro higher concentrations of Aβ oligomers, in the µM range, are well known to induce neuronal death by apoptosis^30,47,48^, lower pM to nM Aβ concentrations have been shown to bidirectionally regulate activity-dependent synaptic plasticity^49^. However, only few studies so far addressed the sublethal Aβ effects on the architecture of neurons. Here we show that Aβ_1–42_ oligomers accumulate at dendritic spines of wild type hippocampal neurons within minutes and reside there up to hours after starting the treatment. Likewise, synthetic Aβ oligomers were previously shown to specifically accumulate at excitatory synapses in an activity-dependent manner^31,50,51^. Since most of the excitatory synapses in the mammalian brain form contacts onto dendritic spines these previous observations are in good agreement with our results. Moreover, we observed a concentration-dependent decrease in dendritic spine density in primary hippocampal neurons treated with synthetic Aβ_1–42_ oligomers. In fact, dendritic spine loss is one of the main hallmarks of AD^52–55^. In addition, we observed that the remaining dendritic spines show an increase in their length and a decrease in the head width, typical of an immature phenotype. These findings are supported by results of previous studies showing a rapid decrease in the density of both synaptic structures and dendritic spine in response to both naturally secreted and synthetic Aβ peptides^27,31,51,56^ and identify a strong and reliable phenotype to be used to analyze the downstream signaling involved in this processes. Importantly, our results show that under the experimental conditions used in this study, the number of cleaved caspase-3 and TUNEL-positive neurons in hippocampal cultures is extremely low and especially it is not increased upon a 6 h treatment with 500 nM Aβ_1–42_ oligomers. This observation strongly indicates that the dendritic spine phenotype observed here is not the consequence of Aβ-induced apoptosis but rather of a sublethal effect of Aβ oligomers.

Several surface receptors have been identified to bind Aβ oligomers at the neuronal surface and trigger a series of intracellular signaling pathways negatively affecting their survival and function^57^. However, the role of several of these receptors is still controversial and their downstream signaling mediating the synaptic effects of Aβ_1–42_ oligomers are largely unknown. We show here strong evidence for a role of the p75^NTR^ in mediating the effects of Aβ_1–42_ oligomers on the neuronal architecture in a specific manner, since a complete p75^NTR^ knockout significantly reduces the accumulation of Aβ_1–42_ to dendritic spines and completely rescues spine loss and changes in spine architecture. Conversely, p75^NTR^ overexpression strengthens both Aβ_1–42_ accumulation and spine loss without exacerbating the changes in dendritic spine architecture observed under control conditions. A link between p75^NTR^ signaling and AD is supported by various observations both in human patients^58,59^ and in mouse models^60,61^. Furthermore, p75^NTR^ has been shown to bind Aβ^12^ including its oligomeric form^22^ and to be required for the Aβ-induced death of primary hippocampal neurons both in vitro and in vivo^16^. Upon treatment with Aβ peptides in the µM range p75^NTR^ has been shown to mediate the activation of downstream signaling cascades involving the activation of JNK^62^ and leading to cell death^12,14,16^. However, whether nM concentrations of Aβ_1–42_ oligomers may activate a different intracellular cascade resulting in changes in neuronal and synaptic structure rather than cell death is not known. Supporting this notion is the observation that low concentrations of Aβ as well as sAPPα have been shown to induce neurite outgrowth in cerebellar and cortical neurons in a p75^NTR^-dependent manner^22,63^. On the other hand, p75^NTR^ has been shown to also function as a signal transducer for neurite outgrowth inhibitory molecules^64^ and to negatively modulate dendritic spine density and their architecture in healthy adult hippocampal neurons^9^ supporting our current observations upon Aβ treatment. However, intracellular signaling linking Aβ interactions with p75^NTR^ to dendritic spine alterations are still unknown. Here we show that Aβ-dependent p75^NTR^ signaling results in the fast activation of RhoA, triggering the activation of ROCK and causing an increase in the F-actin levels within individual spines. These events require p75^NTR^ as they are prevented in p75^NTR^ knockout neurons as well as in neurons treated with the peptide TAT-Pep5, known to act as a downstream signaling silencer by disrupting the recruitment of Rho-GDI to p75^NTR^ and thereby inhibiting the activation of RhoA^8^. In this respect, it is noteworthy that recent studies described the involvement of the ROCK-LIMK pathway in Aβ-induced dendritic spine degeneration^65,66^. Moreover, Aβ-dependent synaptotoxicity in cortical neurons involves a dysregulation of the actin cytoskeleton characterized by a cofilin driven increase in actin stability^67^.

Taken together our observations provide a strong support for a role of p75^NTR^ in mediating the negative effects of Aβ_1–42_ oligomers at dendritic spines and we here specifically describe a downstream signaling pathway that involves RhoA-ROCK activation and regulates the stability of the actin cytoskeleton within spines. Thus, our work contributes to a better understanding of the signaling mechanisms activated upon a treatment with Aβ_1–42_ oligomers and resulting in the typical dendritic spine pathology observed in the early phases of AD and opens new avenues for further treatment option at the very early onset of AD.

## Materials and methods

### Mice

Both wild type (WT) and p75^NTRexonIV^ knockout (p75^NTR^ ko)^29^ mice used in this study were on the C57Bl/6J genetic background. All procedures concerning animals were performed in accordance with the animal welfare representative of the TU Braunschweig and were approved by the LAVES (Oldenburg, Germany, Az. §4 (02.05) TSchB TU BS).

### Cell cultures

Primary hippocampal cultures were prepared from WT or p75^NTR^ KO mice at embryonic day 18 as previously described^68^. Following rapid decapitation, isolated embryo brains were immediately immersed in Gey’s Balanced Salt Solution (GBSS) supplemented with glucose and adjusted to pH 7.3. Under a dissection microscope, the hippocampi were isolated in ice-cold GBSS and their dissociation was achieved by incubation in Trypsin–EDTA at 37 °C for 30 min followed by mechanical dissociation until a single cell suspension was obtained. Subsequently, the cells were re-suspended in NB + medium comprising Neurobasal medium (Gibco) supplemented with 2% B27 (vol/vol), 1% N2 and 0.5 mM Glutamax (Gibco) and plated at a density of 7 × 10^4^/cm^2^ on coverslips (12 mm) or at 450,000 cells/petri dishes (diameter 3 cm), previously coated with poly-L-lysine (Sigma). The cells were incubated at 37 °C with 99% humidity and 5% CO_2_ until usage at DIV14. A medium change was done once a week by exchanging 20% of the medium with fresh NB + medium.

### Transfection

At DIV13 cultured primary hippocampal neurons were transfected using Lipofectamine 2000 (ThermoFisher Scientific) as per manufacturer’s instructions. The following plasmids were used at a concentration of 0.8 μg/well: farnesylated-eGFP (Clonetech: pEGFP-F) alone to visualize the neurons; for overexpression studies rat p75^NTR^ (pcDNA3-ratp75)^9^ was co-expressed with farnesylated-eGFP (f-eGFP) or eGFP-tagged human p75^NTR^ was used alone (p75-eGFP)^33^.

### Aβ peptide preparation

Soluble Aβ_1–42_ oligomers were prepared as previously described^69^. The freeze-dried human Aβ_1–42_ peptides (Bachem) were initially dissolved in sterile Milli-Q water at a stock concentration of 0.5 mM, sonicated in an ice-cold water bath for 10 min, and stored in small aliquots of 4.5 µl at − 70 °C until use. In vitro oligomerizations always were prepared fresh and 1 day prior to the start of a treatment. For this purpose, aliquots were thawed on ice and 3 μg of Aβ_1–42_ were incubated in a final volume of 20 μl sterile PBS at a peptide concentration of 33 µM at 4 °C for 24 h. After this, the oligomerized peptides were diluted into the culture medium (i.e., Neurobasal medium, Gibco) to obtain final treatment concentrations. For negative controls, equivalent amounts of PBS (i.e. vehicle) were used.

### Treatments

24 h post-transfection (DIV14), the cultured hippocampal neurons were treated either with vehicle (PBS) or with Aβ_1–42_ oligomers at varying concentrations: 10 nM, 50 nM, 100 nM, 500 nM. The NB + medium was replaced with fresh Neurobasal medium without supplements and the peptides were diluted directly into the culture wells. The control wells contained no peptides but an equivalent volume of the vehicle used to dilute the oligomerized peptides (PBS). To interfere with p75^NTR^ Rho-ROCK signaling, the cell permeable p75^NTR^ inhibitor TAT-Pep5 Calbiochem (100 nM; Merck Millipore) and the p160 ROCK inhibitor Y-27632 dihydrochloride (10 μM; Tocris Bioscience) were used to pre-treat the neurons for 15 min before the addition of Aβ_1–42_ oligomers into the same culture medium. For the analysis of dendritic spine density and morphology, the cultures were fixed after 15 min or 6 h. For the analysis of the F-actin content as well as for the measurement of RhoA activation the neurons were either fixed or lysed 10 min after Aβ_1–42_ oligomer addition.

### Immunofluorescence

Following a 10 min fixation using cold paraformaldehyde (4% in PBS), the cells were incubated for 1 h at room temperature (RT) in a permeabilizing and blocking buffer containing 0.2% Triton X-100 and 1.5% Normal Goat Serum (NGS, Merck) in PBS. Next, the cells were incubated overnight at 4 °C on a rocker with primary antibodies diluted in the permeabilizing/blocking solution. The following primary antibodies were used: anti-β-Amyloid peptide (either clone BAM-10, Sigma, 1:5,000, or clone 6E10, BioLegend, 1:500); mouse anti-microtubule-associated protein 2 (MAP2, 1:1,000, Sigma); rabbit anti-cleaved caspase-3 (1:500, Millipore). Secondary antibody staining was done in the dark at room temperature for 2 h using the following antibodies: anti-mouse IgGFc_γ_ Subclass-I (1:500, Jackson ImmunoResearch Labs); anti-mouse IgG (H + L) Alexa-fluor 647 (1:500, Jackson ImmunoResearch Labs); anti-rabbit IgG (H + L) Cy3 (1:500, Jackson ImmunoResearch Labs). Finally, the cover slips were washed and mounted onto glass slides using anti-fading Fluoro-Gel embedding medium (Electron Microscopy Sciences). For the visualization of F-actin, some sets of cultures were stained with Alexa-fluor-Phalloidin 647 (ThermoFisher Scientific) diluted 1:40 in PBS following the incubation with the secondary antibody also in the dark for 1 h RT. To minimize actin disruption, the Triton-permeabilization duration was reduced to 15 min. The subsequent steps remained unchanged.

In one set of experiments, DNA fragmentation in dying neurons was detected using the Dead End fluorimetric TUNEL system (Promega) according to the protocol provided. Briefly, after incubation with the secondary antibodies and washes in PBS, the coverslips were incubated in equilibration buffer for 10 min followed by addition of incubation buffer containing terminal deoxynucleaotidyl transferase (TdT) and fluorescein-12-dUTP (nucleotide mix). The coverslips were stained for 60 min at 37 °C in a humidified chamber kept in the dark. The reaction was then stopped using SSC buffer and the coverslips were washed in PBS, counterstained with DAPI, and mounted with anti-fading Fluoro-Gel embedding medium (Electron Microscopy Sciences).

### RhoA G-LISA

The G-LISA RhoA activation assay was performed strictly according to the manufacturer’s instructions (G-LISA activation assay kit, Colorimetric format, Cytoskeleton Inc.). 9 × 10^5^ cells were used for each treatment by pooling cells of two petri dishes (diameter: 3 cm) per treatment. After a 10-min treatment with vehicle or Aβ_1–42_ oligomers, cell lysates were prepared rapidly on ice. The lysates were clarified by centrifugation at 10,000×*g* for 1 min at 4 °C. 30 μl of lysates were used for protein quantification and the remainder was snap-frozen in liquid nitrogen. This entire process was carried out under 10 min and was done sequentially for each test group. Protein quantification was performed by incubating the samples for 1 min at room temperature with the Precision Red Advanced Protein Assay Reagent (Cytoskeleton Inc.) provided by the kit. Absorbance readings were performed with a spectrophotometer at 600 nm. All samples were equilibrated to identical protein concentrations (0.5 mg/ml) and loaded in triplicates onto the plates kept on ice. The samples (in triplicates), the positive RhoA control (duplicate) and the buffer blank (duplicate) were incubated with an anti Rho-antibody and, after multiple washes, with the HRP-conjugated secondary antibody. A color reaction was done by application of freshly prepared HRP A/B detection reagent and the wells were read for absorbance at 490 nm.

### Image acquisition and analysis

Hippocampal neuronal cultures stained for MAP2 and cleaved caspase-3 were imaged using a Zeiss Axioplan2 microscope equipped with a 10 × objective (NA 0.3) and Zeiss AxioCam MRm camera. Different excitation wavelengths were used to image MAP2, cleaved caspase-3, and TUNEL in each field of view. Cleaved caspase-3 and TUNEL-positive neurons were counted manually based on their positivity for MAP2 in merged images created using ImageJ software (https://imagej.nih.gov/ij/).

For dendritic spine analysis, f-eGFP-labeled dendrite stretches were imaged using a confocal laser scanning microscope (Olympus, FluoView1000) equipped with a 40 × objective (oil, NA 1.3) with a 5 × digital zoom for a pixel size of 0.107 µm. Z-stack images were acquired with a 0.35 μm step-size and constant laser intensity. Well defined dendritic segments of healthy neurons, identified by a pyramidal cell body and by the absence of irregular membranous protrusions around the soma, were selected for imaging. Images were then deconvolved using AutoQuant (Media Cybernetics) and imported to the ImageJ software for analyzing dendritic spine density and morphology, Aβ colocalization with spines and Phalloidin fluorescence intensity.

Spine density was determined by analyzing z-stacks containing the entire dendritic stretch. The number of spines per unit of length (µm) was calculated by using the segmented line tool of ImageJ to measure dendritic length and the multipoint selection tool (ImageJ) to count spines. Morphometric analysis of dendritic spines was done in the same z-stacks as for the spine density using the segmented line tool of ImageJ to measure spine length (from its base at the dendrite to its tip) and head width (measured at the widest point of the dendritic spine).

A f-eGFP-labelled spine was considered to be colocalized with Aβ-immunoreactive puncta when seen within the same focal plane or one above or below. For analysis of Phalloidin intensity, regions of interest (ROIs) were drawn around spine heads on merged images, colocalized or not with Aβ-immunoreactivity, while being blinded for the phalloidin channel. Only dendritic spines with clearly defined heads were chosen for this analysis. Additional ROIs were drawn on background (no dendrite and staining). Phalloidin intensities for all selected spine heads were averaged and normalized to the averaged background intensity for each image individually.

$$Roi\frac{avg. \,\, spine}{avg. \,\, background}=Relative \, actin \, intensity$$

All analysis were performed by an experimenter blinded for the genotype and treatment of each sample.

### Data representation and statistical analysis

Data replicates generated from at least three independent experiments were used for statistical analysis. The data were collected and organized in Microsoft Excel. For the generation of graphs and performing the statistical tests, the values were imported into GraphPad Prism 6. Unless otherwise mentioned, all data is reported as mean ± SEM. Student’s t-test was used to compare two independent groups. One-way or Two-way ANOVA multiple-comparisons followed by Sidak or Tukey post-hoc tests, respectively, was used for assessing significance between more than two groups. Significance was considered for *p* value < 0.05; plotting used the notation as *p < 0.05, **p < 0.01, ***p < 0.001, ****p < 0.0001.

## Supplementary information

Supplementary Information.

## Data Availability

All data generated and analyzed during this study are available from the corresponding author on reasonable request.
