# Supplementary material for: Signaling via the p75 neurotrophin receptor facilitates amyloid-β-induced dendritic spine pathology
Source: Sci Rep. 2020 Aug 7;10:13322. doi: 10.1038/s41598-020-70153-4 (PMC7415136; doi:10.1038/s41598-020-70153-4)
Supplement: Supplementary file 1 — Supplementary Information. [file 41598_2020_70153_MOESM1_ESM.pdf]

Supplementary Material for:

Signaling via the p75 neurotrophin receptor facilitates amyloid-β-induced dendritic spine pathology

Abhisarika Patnaik[1], Marta Zagrebelsky[1], Martin Korte[1,2] and Andreas Holz[2*]

Affiliations:

[1] Zoological Institute, Division of Cellular Neurobiology, TU Braunschweig, Braunschweig, D-38108, Germany

[2] Helmholtz Centre for Infection Research, AG NIND, Inhoffenstr. 7, Braunschweig, D-38124, Germany.

[*] Corresponding author:

Andreas Holz, PhD

Group Neuroinflammation and Neurodegeneration
Helmholtz Centre for Infection Research
Inhoffenstrasse 7, D-38124 Braunschweig, Germany

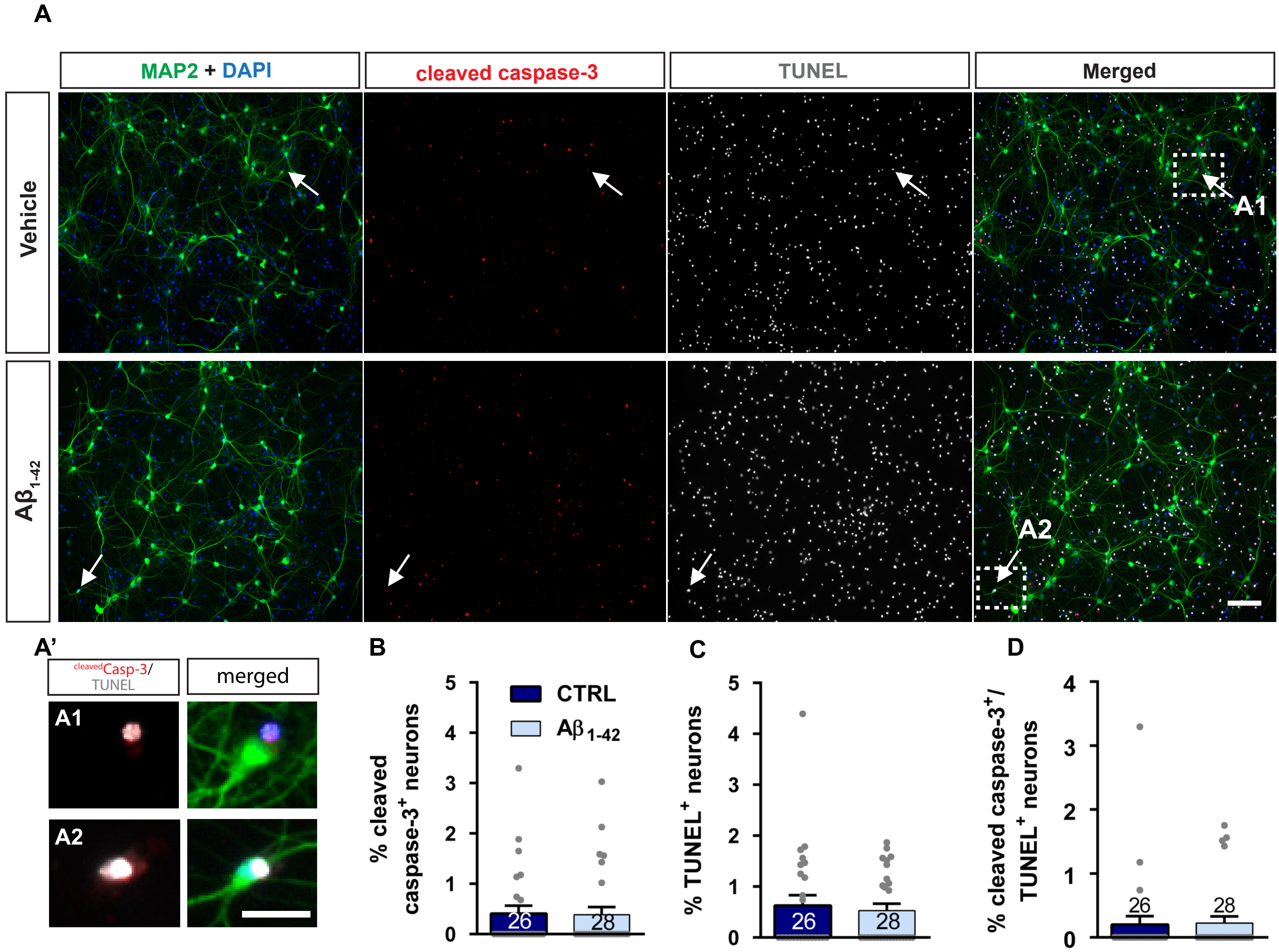

Figure S1: Patnaik et al

Treatment of neuronal cultures with 500 nM amyloid-β oligomers has no significant effect on the number of activated Caspase-3 and TUNEL-positive hippocampal neurons

(A) Shows representative images of primary hippocampal cultures from wild type mice treated for 6 hours with vehicle (top panels) or 500nM $A\beta_{1-42}$ oligomers (bottom panels) and stained for MAP2 (green), cleaved caspase-3 (red) or Tunel (white). Arrows indicate MAP2 positive neurons also positive both for cleaved caspase-3 and Tunel as see in the merged image on the right. Scale bar is 100µm. (A') shows two close ups of MAP2 positive neurons, also positive for both activated caspase-3 and TUNEL in cultures treated with vehicle (above) or 500 nM $A\beta_{1-42}$ oligomers (below, scale bar is 20 µm). The graphs show the proportion in % of cleaved caspase-3- (B), TUNEL- (C) or both cleaved caspase-3 and TUNEL-positive neurons (D) for cultures treated with vehicle (dark blue) or 500 nM $A\beta_{1-42}$ oligomers (light blue). The numbers on top of the bars in the graphs indicate the number of fields of views used for the analysis. The bars reflect mean values + SEM. Statistical significance was tested using a Student's t-test. Note that while in the images many non-neuronal cells are positive for activated caspase-3 and TUNEL, the number of dying neurons is extremely low and not significantly different between the two conditions.

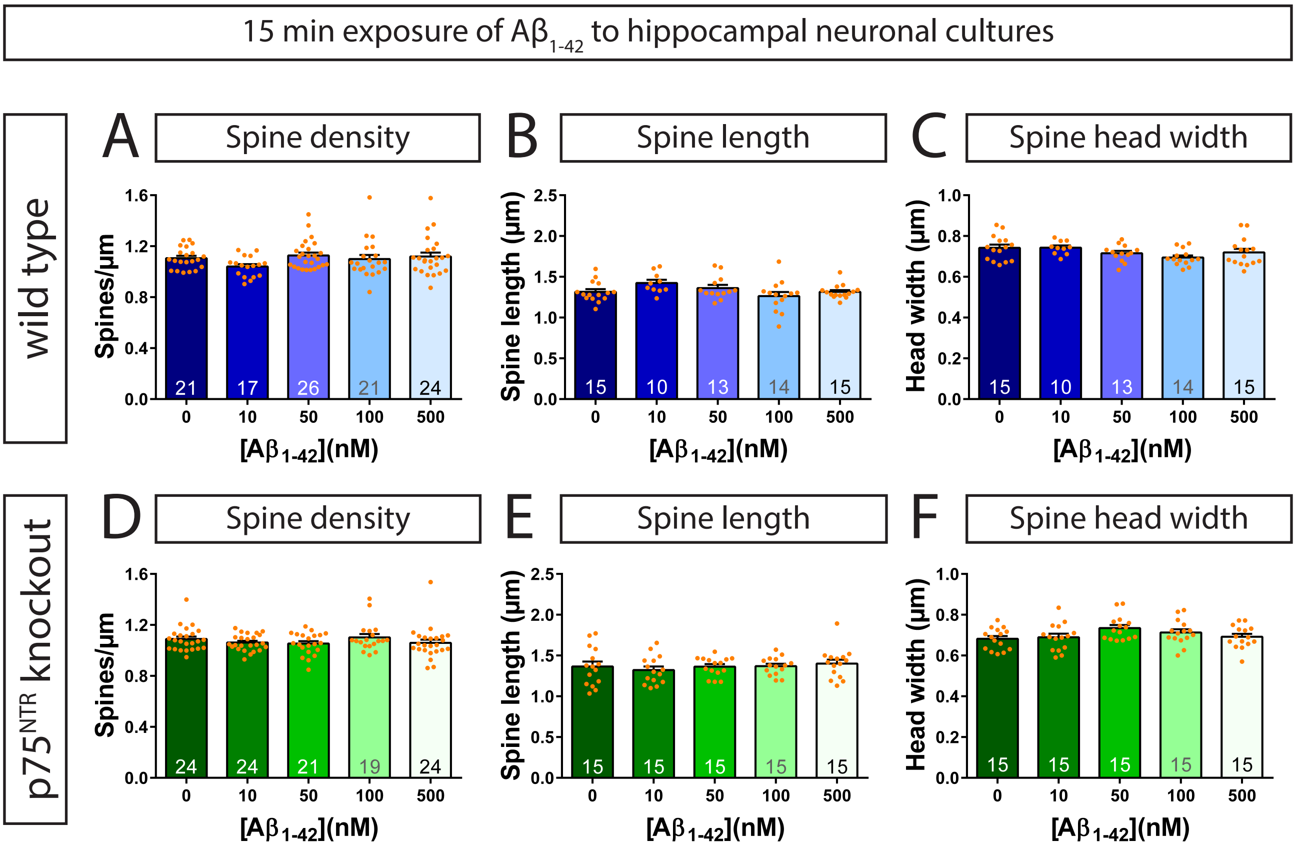

Figure S2: Patnaik et al

Brief treatment of neuronal cultures with amyloid-β oligomers has no impact on dendritic spine density and morphology

Primary hippocampal neurons from wild type (A-C) and $p75^{NTR}$ ko (D-F) mice either were incubated with vehicle (0) or were treated for 15 minutes with 10, 50, 100, or 500 nM aggregated $A\beta_{1-42}$ peptides. Spine density (A, D), spine length (B, E), and spine head width (C, F) were determined by analyzing dendritic segments (each with a length of at least 100 µm). Bars reflect mean values + SEM. Number of evaluated neurons from three independent experiments is printed for each column of the graph. To test for statistical significance a One-way ANOVA with Sidak post-test was employed. Note that comparing the means of the analyzed spine parameters of amyloid-β-treated to their corresponding vehicle control groups never revealed any statistical significance.
